# Supplementary material for: Antiparasitic Potential of Chromatographic Fractions of Nephrolepis biserrata and Liquid Chromatography-Quadrupole Time-of-Flight-Mass Spectrometry Analysis
Source: Molecules. 2021 Jan 19;26(2):499. doi: 10.3390/molecules26020499 (PMC7832296; doi:10.3390/molecules26020499)
Supplement: Supplementary file 1 [file molecules-26-00499-s001.pdf]

Supplementary data

Table S1: Data of death time and percentage of the fractions of *N. biserrata* against the marine leeches in replicates

		Replicates 1-3	5 leeches per Group				
**Groups**	**No of Parasites**	**Death Time Experiment 1**	**Death Time Experiment 2**	**Death Time Experiment 3**	**Average Mean**	**Average SD**	**Death %**
**Control**	1	0	0	0			0
	2	0	0	0			0
	3	0	0	0			0
	4	0	0	0			0
	5	0	0	0			0
	**Mean**	0	0	0	0		
	**SD**	0	0	0		0	
**Formalin (0.02%) (v/v)**	1	3.4	3	3.44			100
	2	3.4	4	3.55			100
	3	3.46	3.44	4			100
	4	6	5.4	4.55			100
	5	3.5	4	3.4			100
	**Mean**	3.952	3.968	3.788	**3.902666667**		
	**SD**	1.145652652	0.903946901	0.488333902		**0.845977819**	

***Nephrolepis biserrata* Fraction1 (NB F1) (4.80 mg/ml)**	1	0	0	0			0
	2	0	0	0			0
	3	0	0	0			0
	4	0	0	0			0
	5	0	0	0			0
	**Mean**	0	0	0	0		
	**SD**	0	0	0		0	
NB F2 (20 mg/ml)	1	0	0	0			0
	2	0	0	0			0
	3	0	0	0			0
	4	0	0	0			0
	5	0	0	0			0
	**Mean**	0	0	0	0		
	**SD**	0	0	0		0	
NB F3 (2.50 mg/ml)	1	1.4	1.15	1.45			100
	2	3	1.3	2			100
	3	2.3	1.41	3			100
	4	2.14	2.07	1.15			100
	5	3	2.07	2.07			100
	**Mean**	2.368	1.4825	1.934	**1.928166667**		
	**SD**	0.669417657	0.40590434	0.708540754		**0.594620917**	

NB F4 (14 mg/ml)	1	30.5	30	32.5			100
	2	32.29	33	33.3			100
	3	34	36.9	36.4			100
	4	38	40	38			100
	5	38	34.5	39.4			100
	**Mean**	33.6975	34.975	35.05	**34.57416667**		
	**SD**	3.204583956	4.382065723	2.587791851		**3.39148051**	
NB F5 (15.30 mg/ml)	1	31	33	32.2			100
	2	37.17	35	34.5			100
	3	37.2	39.9	34.6			100
	4	43.11	41.2	43			100
	5	43.35	42	43.7			100
	**Mean**	37.12	37.275	36.075	**36.82333333**		
	**SD**	4.94447166	3.905018139	4.74789427		**4.532461357**	
NB F6 (17.20 mg/ml)	1	0	0	0			0
	2	0	0	0			0
	3	0	0	0			0
	4	0	0	0			0
	5	0	0	0			0
	**Mean**	0	0	0	**0**		
	**SD**	0	0	0		**0**	

NB F7 (20 mg/ml)	1	0	0	0			0
	2	0	0	0			0
	3	0	0	0			0
	4	0	0	0			0
	5	0	0	0			0
	**Mean**	0	0	0	**0**		
	**SD**	0	0	0		**0**	
